# Information Loss in Harmonizing Granular Race and Ethnicity Data: Descriptive Study of Standards

**DOI:** 10.2196/14591

**Published:** 2020-07-20

**Authors:** Karen Wang, Holly Grossetta Nardini, Lori Post, Todd Edwards, Marcella Nunez-Smith, Cynthia Brandt

**Affiliations:** 1 Equity Research and Innovation Center General Internal Medicine Yale School of Medicine New Haven, CT United States; 2 Center for Medical Informatics Yale School of Medicine New Haven, CT United States; 3 Harvey Cushing/John Hay Whitney Medical Library Yale School of Medicine New Haven, CT United States; 4 Buehler Center for Health Policy and Economics Feinberg School of Medicine Chicago, IL United States; 5 Division of Epidemiology Department of Medicine Vanderbilt Genetics Institute, Vanderbilt University Medical Center Nashville, TN United States; 6 Veteran Affairs Connecticut Healthcare System US Department of Veteran Affairs West Haven, CT United States

**Keywords:** continental population groups, multiracial populations, multiethnic groups, data standards, health status disparities, race factors, demography

## Abstract

**Background:**

Data standards for race and ethnicity have significant implications for health equity research.

**Objective:**

We aim to describe a challenge encountered when working with a multiple–race and ethnicity assessment in the Eastern Caribbean Health Outcomes Research Network (ECHORN), a research collaborative of Barbados, Puerto Rico, Trinidad and Tobago, and the US Virgin Islands.

**Methods:**

We examined the data standards guiding harmonization of race and ethnicity data for multiracial and multiethnic populations, using the Office of Management and Budget (OMB) Statistical Policy Directive No. 15.

**Results:**

Of 1211 participants in the ECHORN cohort study, 901 (74.40%) selected 1 racial category. Of those that selected 1 category, 13.0% (117/901) selected Caribbean; 6.4% (58/901), Puerto Rican or Boricua; and 13.5% (122/901), the mixed or multiracial category. A total of 17.84% (216/1211) of participants selected 2 or more categories, with 15.19% (184/1211) selecting 2 categories and 2.64% (32/1211) selecting 3 or more categories. With aggregation of ECHORN data into OMB categories, 27.91% (338/1211) of the participants can be placed in the “more than one race” category.

**Conclusions:**

This analysis exposes the fundamental informatics challenges that current race and ethnicity data standards present to meaningful collection, organization, and dissemination of granular data about subgroup populations in diverse and marginalized communities. Current standards should reflect the science of measuring race and ethnicity and the need for multidisciplinary teams to improve evolving standards throughout the data life cycle.

## Introduction

The cutting edge of precision medicine, such as the integration of contextual data about the social determinants of health with individual health data and the leveraging of data from across different studies, is seen as a mechanism to innovate and solve health problems for all populations [1,2]. These solutions can only be realized by grounding them in the concept of health equity [3,4]. According to the Robert Wood Johnson Foundation [5],

Health equity means that everyone has a fair and just opportunity to be healthier. This requires removing obstacles to health such as poverty, discrimination, and their consequences, including powerlessness and lack of access to good jobs with fair pay, quality education and housing, safe environments, and health care.

To attain health equity, the research community must identify where health disparities exist but may inadvertently exacerbate health disparities by failing to identify invisible or at-risk populations. Without this information, we may reinforce inequities rather than identify policies, laws, systems, environments, and practices that can improve opportunities for health in these communities [6-9].

There is a significant body of literature about the collection of and proposed definitions for more granular data for race, ethnicity, and other demographic data in the health sciences that would better identify at-risk populations. In some areas, more granular data collection is used to be more inclusive of the ways that individuals self-identify and to examine how racism and discrimination affect health in these populations [10-13]. However, the research community has not sufficiently considered how to meaningfully organize granular data about subgroup populations in diverse communities so that these data can be used across multiple studies to address the challenges in these communities [5,14]. In this paper, we used our experience with a cohort study in the eastern Caribbean to illustrate the need for an updated and comprehensive data standard to enable future data integration and sharing of data sources for diverse multiracial populations.

## Methods

The Eastern Caribbean Health Outcomes Research Network (ECHORN) cohort study follows community-dwelling adults older than 40 years residing in Barbados, Trinidad, Tobago, and the United States’ territories of the US Virgin Islands and Puerto Rico [15]. Baseline participants were enrolled between 2013 and 2016 and completed a questionnaire to capture self-reported sociodemographics and health-related information. In the questionnaire, participants had the option to self-identify race and ethnicity by selecting any of the items listed: mixed or multiracial, white, black or African, Caribbean, Asian, East Indian, Hispanic or Latino, Puerto Rican or Boricua, other, or prefer not to answer. This list was developed with stakeholder input to reflect the current scientific literature on the measurement of race and ethnicity in epidemiologic and health outcomes research. Excluding the choice of “other”, there were 256 potential answers.

The National Institutes of Health (NIH) requires investigators to collect and report race and ethnicity based on Office of Management and Budget (OMB) Statistical Policy Directive No. 15 data standards, developed in 1977 and updated in 2007 [16]. The standards include 7 race and ethnicity categories, with instructions to use 2 questions. The first question collects ethnicity information (Hispanic/Latino or not Hispanic/Latino), and the second provides the option to select more than one of the 5 racial categories (American Indian or Alaska Native, Asian, black or African American, Native Hawaiian or other Pacific Islander, or white). Data reported are required to include (1) the number of respondents in each ethnic category, (2) the number of respondents who selected only 1 of 5 racial categories, (3) the number of respondents who selected multiple racial categories, and (4) the number of respondents in each racial category who identified as Hispanic or Latino. Reporting detailed distributions of multiple responses should be aggregated into the required categories.

## Results

In an analysis of ECHORN data from 1211 participants, 901 (74.40%) selected 1 racial category. Of those that selected only 1 category, 13.0% (117/901) selected Caribbean; 6.4% (58/901), Puerto Rican or Boricua; and 13.5% (122/901), mixed or multiracial. A total of 17.84% (216/1211) of participants selected 2 or more categories, with 15.19% (184/1211) selecting 2 categories and 2.64% (32/1211) selecting 3 or more categories. The participants who selected more than one category included but were not limited to white Hispanic, black Hispanic, black Asian, black East Indian, and Caribbean Hispanic individuals. Based on OMB data standards, by collapsing those who selected multiple groups and those who selected only the multiracial and mixed category, 27.91% (338/1211) of the ECHORN participants are placed in the “more than one race” category. The nuanced race and ethnicity data become simply “more than one race, black or African American, white, Hispanic or Latino, Asian, or not reported/unknown.”

## Discussion

### Principal Findings

Data standards for race and ethnicity need to reflect and evolve with the scientific advances around the measurement and mechanisms by which race and ethnicity can affect health [17,18]. The stated goals of the OMB standards are to harmonize the collection and presentation of population race and ethnicity information across federal databases and to facilitate comparisons and analyses [16]. To achieve the objectives of precision medicine, ECHORN data on those who selected multiple categories, such as the white Hispanic, black Hispanic, black Asian, black East Indian, or Caribbean Hispanic individuals, could be aggregated with other data sources with defined granular race and ethnicity data on this population. For example, the US Census Bureau has developed granular data collection standards on race and ethnicity, quantifying the growth of these multiracial and multiethnic populations. The State of Massachusetts, along with other states, has for several decades mandated the collection of more granular race and ethnicity information, with over 30 categories on many data collection forms, including birth certificates and clinical data [19-21]. However, it is not specified how multiracial and multiethnic populations’ granular data can be harmonized to facilitate comparisons and analyses across any datasets collected using different standards. Current research collaborations, such as Observational Health Data Sciences and Informatics, that are focused on facilitating harmonization and sharing data by using standard ontologies such as Systematized Nomenclature of Medicine - Clinical Terms and Logical Observation Identifiers Names and Codes, have no agreed-upon standard for granular race and ethnicity data or a mechanism to map across data standards [22].

Standards have not been refined or expanded adequately to accommodate studies collecting granular race and ethnicity data on mixed or multiracial individuals [23,24], and these imprecise standards have unmeasured effects on health. Current standards may contribute to health disparities by providing insights on persons privileged by these data structures and by further marginalizing persons whose racial and ethnic identity is obscured in suboptimal data standards for classification and harmonization. While the OMB standards clearly state that “the racial and ethnic categories set forth in the standards should not be interpreted as being primarily biological or genetic in reference,” [16] current standards used in health sciences continue to support the conflation of the social categories of race and ethnicity with the biologic and genetic categories in day-to-day practice. This conflation has powerful implications for population-level biomedical discoveries and clinical care and treatment [25]. For example, inherent inequities in data standards are reflected in the continued use of monoracial guidelines as the standard of care in clinical practice, in spite of knowledge that suggests the fallacies of this framework [26,27]. It also has implications for population genetics and the translation of discoveries. These social constructs are used to develop standards for genetic population norms, wherein outlier data are discarded and study findings are associated with a singular racial group, influencing the development of biologic treatments directed at mutations that are predominantly in a particular racial group [28,29]. However, there is no biological origin for much of what we define as health inequities [30]. At the individual level, understanding how contextual factors, such as racism and discrimination, affect how a multiracial and multiethnic person understands and addresses their own health risks is significantly limited because of the lack of data and data standards that organize and share granular data in a viable way [31]. Importantly, multiracial and multiethnic populations are only one example of diverse subgroups affected by this lack of evolved standards; we risk the loss of rich information on the individuals who select more than one category for their race and ethnicity.

Several incremental changes can be instituted to improve these data standards on race and ethnicity. In addition to standardizing the definitions and collection of more granular race and ethnicity data across research studies, as advocated by numerous prior researchers [10-13], the standards need to be developed and adopted by funding agencies, such as the NIH, so that researchers from different groups who are collecting granular data can share and aggregate data [32,33]. These comprehensive data standards need to specify how to systematically categorize self-identified granular race and ethnicity data for those who have selected 2 or more categories. The standards can provide mechanisms to map race and ethnicity data collection standards to other standards over time, such as the Centers for Disease Control and Prevention Race and Ethnicity Code Set, Version 1.0, or the United States Census Bureau [32-34]. As data are collected and shared between regional and national entities, this mapping between standards will enable the aggregation of smaller data about underrepresented populations across studies and global contexts [32,35,36] and will provide a systematic method for individuals who have collected granular data to organize and map their data to less-evolved data collection standards.

### Conclusion

Comprehensive data standards for race and ethnicity throughout the life course of health science research studies are critical to identifying and achieving heath equity for populations differentially affected by discrimination [23,24,37]. Partnerships with relevant stakeholders in the development and mapping of standards are essential and will likely increase availability of meaningful and usable data [38]. Multidisciplinary research teams that focus on health equity (eg, informaticists, data scientists, information scientists, geneticists, sociologists, and public health researchers) need to adapt standards and advance the theory and systems for organizing, storing, and analyzing complex data. These standards are urgently needed to ensure the sustained value of data collected about diverse populations and their health. With the status quo, we will continue to privilege one group’s data over another’s, lack meaningful and useable information of diverse racial and ethnic population groups, and likely further perpetuate health inequities.

## Abbreviations

ECHORN: Eastern Caribbean Health Outcomes Research Network
NIH: National Institutes of Health
OMB: Office of Management and Budget
